# Novel Fast-Setting and Mechanically-Strong Calcium Phosphate Pulp-Capping Cement with Metformin Release to Enhance Dental Pulp Stem Cells

**DOI:** 10.3390/bioengineering12010013

**Published:** 2024-12-28

**Authors:** Mohammad Alenizy, Abdullah Alhussein, Nader Almutairi, Ibrahim Ba-Armah, Tao Ma, Suping Wang, Nageswara R. Pilli, Maureen A. Kane, Michael D. Weir, Abraham Schneider, Hockin H. K. Xu

**Affiliations:** 1 Dental Biomedical Sciences Ph.D. Program, Graduate School, University of Maryland, Baltimore, MD 21201, USA; malenizy@umaryland.edu (M.A.);; 2 Department of Biomaterials and Regenerative Dental Medicine, University Maryland School of Dentistry, Baltimore, MD 21201, USA; 3 Department of Restorative Dental Sciences, University of Hail, Hail 55475, Saudi Arabia; 4 Department of Restorative Dental Science, College of Dentistry, King Saud University, P.O. Box 60169, Riyadh 11545, Saudi Arabia; 5 Department of Conservative Dental Sciences, College of Dentistry, Prince Sattam bin Abdulaziz University, Alkharj 11942, Saudi Arabia; 6 Department of Restorative Dental Sciences, College of Dentistry, Imam Abdulrahman Bin Faisal University, Dammam 31441, Saudi Arabia; 7 Department of Oncology and Diagnostic Sciences, School of Dentistry, University of Maryland, Baltimore, MD 21201, USA; 8 Stomatology Center, The First Affiliated Hospital of Zhengzhou University, Zhengzhou 450052, China; 9 Department of Pharmaceutical Sciences, School of Pharmacy Mass Spectrometry Center, University of Maryland, Baltimore, MD 21201, USA; 10 Marlene and Stewart Greenebaum Cancer Center, School of Medicine, University of Maryland, Baltimore, MD 21201, USA; 11 Center for Stem Cell Biology & Regenerative Medicine, School of Medicine, University of Maryland, Baltimore, MD 21201, USA

**Keywords:** dentin regeneration, direct pulp capping, bioactive material, chitosan, metformin, biocompatibility

## Abstract

Traditional pulp-capping materials like mineral trioxide aggregate (MTA) offer excellent biocompatibility and sealing, but limitations such as prolonged setting time, low bioactivity, and high costs persist. Metformin, with its potential in craniofacial regeneration, could enhance dentin synthesis by targeting pulp cells. This study aimed to: (1) develop a calcium phosphate cement with chitosan (CPCC) with improved physio-mechanical properties; (2) incorporate metformin (CPCC-Met) to assess release; and (3) evaluate human dental pulp stem cells (hDPSCs) response. CPCC was mixed at different powder-to-liquid ratios to evaluate physio-mechanical properties compared to MTA. The optimized CPCC formulation was loaded with 0, 50, 100, and 150 µg of metformin to measure release and assess hDPSCs attachment and proliferation (1, 4, and 7 d) via live/dead imaging and SEM. One-way ANOVA was used for statistical analysis. Results showed CPCC at a 3.25:1 ratio significantly reduced setting time to 41.5 min versus 123 min for MTA (*p* < 0.05). Metformin release correlated with concentration, and SEM confirmed the presence of a porous, hydroxyapatite-rich surface. Cell viability was consistently high across groups (>93% at 1 d, >95% at 4 d, ≈98% at 7 d), with no significant differences (*p* > 0.05). These findings suggest that the novel CPCC-Met demonstrates promise as a fast-setting, cost-effective pulp-capping material, offering metformin delivery to enhance dentin repair.

## 1. Introduction

The integrity of the dentin-pulp complex in teeth may become compromised when the vital pulp is exposed, from deep caries removal, traumatic injuries, or accidentally during restorative procedures. Preserving pulp vitality through direct pulp capping supports optimal tooth development, preventing the need for more invasive and expensive treatments.

Direct pulp capping involves the placement of a biocompatible material directly onto the exposed pulp to preserve pulp vitality via mineralized tissue formation. The paramount feature of direct pulp capping materials lies in their exceptional biocompatibility [1]. Historically, calcium hydroxide has been considered the gold standard material for direct pulp capping due to its potent antimicrobial properties [2,3,4]. It induces chemical injury through hydroxyl ions, causing superficial necrosis that stimulates the pulp to form a reparative dentin bridge, which is crucial for treatment success. However, this bridge formation often contains tunnel defects, compromising its effectiveness as a barrier against bacterial infection. Additionally, calcium hydroxide is prone to dissolution, creating dead spaces that lead to microleakage [3].

Presently, the utilization of MTA has become more common, demonstrating highly favorable clinical and in vitro outcomes [5]. Research findings suggest it exhibits outstanding biocompatibility and sealing performance and is linked with positive clinical outcomes [6]. However, MTA has inherent drawbacks that hinder clinicians’ utilization, such as prolonged setting times, high costs, handling difficulties, and undesirable discoloration [7,8,9]. In addition, a key component of MTA is bismuth oxide, which dissolves in acidic environments. This is a concern because when MTA is used in an acidic environment, such as inflamed tissues, it releases bismuth oxide. This release increases the rate of discoloration and jeopardizes MTA’s biocompatibility because bismuth oxide does not support cell growth in cell culture [10,11].

Thus, targeting pulp cells in dentin synthesis, using innovative and cost-effective bioactive formulations with enhanced physio-mechanical characteristics, holds promise for yielding genuinely advantageous and economically efficient therapeutic results.

Research into calcium phosphate compounds for bone repair dates back to 1920, yet their clinical application remained limited until the 1980s when self-setting calcium phosphate cement (CPC) was developed [12]. Commercial CPC products were initially launched in the 1990s to treat craniofacial defects [13]. CPC shows great promise in craniofacial regeneration, especially for complex bone defects. Its bioactivity and flexibility to be molded into irregular shapes make it a strong material choice for craniofacial applications. Research indicates that macroporous CPC supports bone formation and blood vessel growth in cranial defect models, promoting new bone integration within the defect over time [14,15,16]. CPC demonstrates exceptional biocompatibility due to the formation of brushite (at pH < 4.2) and apatite, hydroxyapatite (HA) or calcium-deficient HA (at pH > 4.2) as the final products [17]. Moreover, nano amorphous calcium phosphate has been shown to significantly increase tertiary dentin thickness when used to restore rat first molars with no direct pulp contact [18]. CPC is widely recognized for its cost-effectiveness and biocompatibility, making it a promising candidate for dental applications. Recent research suggests that incorporating chitosan into CPC formulations can enhance its mechanical strength, improve durability, and reduce setting time [19].

Metformin is a safe, non-toxic medication for lowering blood sugar. It is now advocated as the primary oral treatment for type 2 diabetes mellitus and belongs to the biguanide family [20]. Its primary action involves acutely reducing glucose production in the liver by mildly and temporarily inhibiting the mitochondrial respiratory chain complex I. Moreover, the subsequent decrease in liver energy levels triggers the activation of AMP-activated protein kinase (AMPK), a cellular metabolic regulator, which is widely recognized as the mechanism underlying metformin’s effects on liver glucose production [21,22]. Furthermore, metformin has been shown in multiple studies to stimulate odontoblastic differentiation and mineral synthesis in dental pulp stem cells (DPSCs) and has yielded excellent results in craniofacial regeneration [23,24,25].

Adding metformin to CPC formulation has demonstrated potential in promoting cell differentiation and inducing mineralized tissue formation, as shown in previous studies from our group [19,25,26,27,28], which have focused explicitly on its osteogenic effects and sustained drug release capabilities within CPC. These findings underscore metformin’s ability to enhance the biological performance of CPC in pulp capping by supporting cell proliferation and mineralization. By integrating metformin into a CPCC matrix, a novel direct pulp capping material could be developed to deliver localized metformin to the pulp tissue while providing the necessary structural and biological support for dentin regeneration.

This study aimed to: (1) develop CPCC with enhanced physio-mechanical characteristics to overcome MTA’s drawbacks; (2) incorporate and measure metformin release; and (3) explore the effect of CPCC-Met on the attachment and proliferation of hDPSCs. The following hypotheses were tested: (1) the novel CPCC would exhibit superior physio-mechanical properties compared to MTA; (2) the incorporation of different Metformin concentrations into CPCC-Met would yield to unequal amount of released metformin from the cement matrix; and (3) CPCC-Met would positively support the attachment and proliferation of hDPSCs similar to CPCC without metformin and MTA.

## 2. Materials and Methods

### 2.1. Preparation of CPCC Cement

CPC powder is comprised of tetracalcium phosphate (TTCP; Ca4(PO4)2O) and dicalcium phosphate anhydrous (DCPA; CaHPO4) [19,29]. TTCP was synthesized through a solid-state reaction using an equimolar amount of DCPA and calcium carbonate (both from J. T. Baker, Phillipsburg, NJ, USA). The mixture was heated at 1500 °C for 6 h in a furnace (LHTCT, Nabertherm, New Castle, DE, USA), then rapidly cooled to room temperature within a desiccator. The resulting mixture was ground and sieved to achieve an average particle size of 17 µm. DCPA was ground for 24 h in a planetary ball mill model PM-100 (Retsch, Haan Mettman, Germany) with 95% ethanol to produce a powder with an average particle size of 1 μm. The CPC powder was created by mixing TTCP and DCPA in a molar ratio of 1:1. The liquid chitosan malate was prepared by dissolving high molecular weight chitosan 800–2000 cps (Sigma Aldrich, St. Louis, MO, USA) in a 60% mass concentration of malic acid (Sigma Aldrich, St. Louis, MO, USA) and then lyophilized for future use. The chitosan solution was employed as the liquid component for CPCC, with a chitosan/(chitosan + water) mass fraction of 10%.

### 2.2. Mechanical and Physical Properties Evaluation

To examine the impact of the P:L ratio on the physical and mechanical properties, CPCC specimens were divided into six groups, each representing different P:L ratios ranging from 2:1 to 4:1. In addition, one group was allocated for the inclusion of a commercially available MTA (ProRoot MTA, Dentsply Sirona, Tulsa, OK, USA):

- CPC 2:1 + 10% chitosan (CPCC-2);
- CPC 2.5:1 + 10% chitosan (CPCC-2.5);
- CPC 3:1 + 10% chitosan (CPCC-3);
- CPC 3.25:1 + 10% chitosan (CPCC-3.25);
- CPC 3.5:1 + 10% chitosan (CPCC-3.5);
- CPC 4:1 + 10% chitosan (CPCC-4);
- MTA (ProRoot MTA, Dentsply Sirona, Tulsa, OK, USA).

#### 2.2.1. Flexural Strength, Elastic Modulus, and Work-of-Fracture

The CPCC bars were produced using a stainless steel mold with dimensions of 4 × 3 × 25 mm. The molds were filled and subsequently placed in a humidor with 100% humidity at 37 °C for 4 h before demolding. MTA specimens for mechanical testing were prepared by mixing the powder and liquid according to the manufacturer’s instructions. Afterward, all samples were immersed in distilled water at 37 °C for 24 h prior to testing.

The flexural strength, elastic modulus, and work-of-fracture (n = 6) were determined through a 3-point flexural test utilizing a 20 mm span and a crosshead speed of 1 mm/min on a computer-controlled Universal Testing Machine (MTS, Insight 1, Cary, NC, USA).

Work-of-fracture, or toughness, is the energy required to fracture the specimen. This was obtained from the area under the load-displacement curve divided by the specimen’s cross-sectional area [30]. The test was stopped at a maximum crosshead displacement of 1 mm for a consistent calculation of work-of-fracture.

Flexural strength was calculated by the below equation where *Fmax* is the maximum load at fracture, in Newtons; *L* is the distance in mm between the supports; *b* is the width of the specimen; and *h* is the height.

$$S=\frac{3\times Fmax\times L}{2\times b\times h^{2}}$$

Elastic modulus was calculated using the equation below where the slope of the load-displacement curve in the linear elastic area is determined by dividing the load *F* by the corresponding displacement *C.*

$$E=(\frac{F}{C})\times (\frac{L^{3}}{4\times b\times h^{2}})$$

#### 2.2.2. Flowability

The samples were prepared and tested following the specifications outlined in ISO 6876 [31]. The materials (n = 3) were mixed on a glass slab using a stainless steel spatula until achieving a uniform consistency, and 0.5 mL of each was dispensed onto the center of a polished glass slab measuring 40 × 40 mm. Another glass plate was then centrally positioned over the material, followed by a weight totaling 120 g. Ten minutes after mixing, the weight was removed, and the maximum and minimum diameters of the compressed material discs were measured using a digital caliper. Two conditions were necessary to validate the tests: the difference between the maximum and minimum diameters could not exceed 1.0 mm, and the compressed material had to maintain a uniform shape.

#### 2.2.3. Setting Time

The setting time was assessed using the Gilmore needle method (n = 3) following the guidelines outlined in the ASTM standard C266-03 [32] (American Society for Testing and Materials [ASTM] International, West Conshohocken, PA, USA) [33]. The ultimate setting time refers to the duration required for the test specimen to support a heavier Gilmore needle, which weighs 453.6 g and has a tip diameter of 1.06 mm without displaying noticeable indentation.

### 2.3. Metformin Release

Based on the results from the mechanical and physical properties testing, the CPCC3.25 formulation was selected as the optimized composition due to its desirable physio-mechanical properties. Metformin was incorporated into this formulation by adding the specified mass of metformin into the liquid chitosan before mixing. This allowed for the measurement of metformin release and the investigation of its effects on the attachment and proliferation of hDPSCs. The disks were divided into four groups based on metformin loading mass per disk: 0 µg (CPCC-0), 50 µg (CPCC-Met-50), 100 µg (CPCC-Met-100), and 150 µg (CPCC-Met-150) of metformin.

Metformin release from the CPCC-Met disks (d = 8 mm, t = 1 mm; n = 4) was assessed by placing disks from each group in an air-tight vial, each filled with 1 mL of deionized water. The disks were transferred to fresh vials every 24 h from day one until day 7 and then every seven days until day 28. The aliquots collected from each well on days 1, 2, 3, 4, 5, 6, 7, 14, 21, and 28 were then analyzed for metformin release using high-performance liquid chromatography and mass spectrometry (LC-MS/MS) [34]. LC-MS/MS analysis was performed on a TSQ Altis Triple Stage Quadrupole Mass Spectrometer coupled to an Ultimate 3000 RS Liquid Chromatogram system (Thermo Scientific, San Jose, CA, USA). Data collection and analysis were performed using Xcalibur V 2.1 (Thermo Scientific, San Jose, CA, USA).

The mass spectrometer was operated in a positive ion mode, with m/z values of 130.0 → 60.0 for metformin and m/z 136.0 → 60.1 for metformin_d6 (internal standard) using electrospray ionization (ESI) source. Metformin was quantified in the concentration range of 5–5000 ng/mL. The samples were diluted with water and acetonitrile (50:50, *v*/*v*) and were chromatographed on a Zorbax SB C18 (4.6 × 100 mm, 3.5 µm; Agilent Technologies, Santa Clara, CA, USA) column using an isocratic mobile phase consisting of a mixture of 0.1% formic acid in water and acetonitrile (30:70, *v*/*v*) and delivered at a flow rate of 0.6 mL/min.

### 2.4. Cell Viability and Proliferation Assays

In this experiment, we aimed to assess the impact of incorporating metformin into CPCC on hDPSC (Lonza, Walkersville, MD, USA) viability and proliferation compared to MTA. To facilitate a comprehensive analysis, we established five groups:

- Experimental control group: CPCC-3.25 + no metformin (CPCC-0);
- CPCC-3.25 + 50 µg metformin (CPCC-Met-50);
- CPCC-3.25 + 100 µg metformin (CPCC-Met-100);
- CPCC-3.25 + 150 µg metformin (CPCC-Met-150);
- Commercial control group: MTA (ProRoot MTA, Dentsply Sirona, Tulsa, OK, USA) (MTA).

Cell attachment and proliferation within these groups were evaluated using live/dead staining techniques, which allowed us to compare the effects of each treatment on hDPSCs. Disks were seeded with hDPSCs in a 48-well plate at a density of 2.5 × 10^4^ cells per well in 1 mL of human dental pulp stem cell basal medium (Lonza, MD, USA) containing mesenchymal cell growth supplement 10% (MCGS); 2% L-glutamine, 1% ascorbic acid, and 0.1% gentamicin sulfate-amphotericin (GA). At 1, 4 and 7 days, disks were rinsed with PBS and treated with a live/dead staining solution (Invitrogen, Carlsbad, CA, USA) at 37 °C for 15 min, containing 4 mM calcein AM and 2 mM ethidium homodimer-1. Calcein AM is used to stain live cells by producing a green fluorescence by enzymatic conversion in metabolically active cells while ethidium homodimer-1 is used to stain dead cells by emitting red fluorescence when it attaches to DNA in cells with damaged membranes. Samples were then observed via fluorescence microscopy (Cytation 5, Biotek, Santa Clara, CA, USA). Images were captured at three random locations on each disk, resulting in 9 images for each group at every given time (n = 3). Live cell density and viability were calculated by analyzing the fluorescence images using ImageJ software, where the number of green fluorescent cells were counted. The percentage of viable cells (VC%) was determined by using the following formula: VC% = ((Live cell)/(Live cell + Dead cell)) × 100.

### 2.5. Scanning Electron Microscopy (SEM)

SEM (Quanta 200, FEI, Hillsboro, OR, USA) was utilized to analyze the surface and internal morphology of CPCC disks, as well as to observe the adhesion of hDPSCs at 24 h on CPCC-0 and CPCC-Met disks (CPCC-Met-50, CPCC-Met-100, and CPCC-Met-150). Samples were prepared by rinsing with PBS, followed by fixation in 1% glutaraldehyde at 4 °C overnight. Subsequently, the samples were rinsed again with PBS and dehydrated in a graded ethanol treatment. After dehydration, they were rinsed with hexamethyldisilazane and left to air dry overnight. The dried samples were then sputter-coated with platinum before examination under SEM.

### 2.6. Statistical Analysis

Statistical analyses, including normality assessment via the Shapiro–Wilk test, were conducted using Sigma Plot software (Version 12.0; SYSTAT, Chicago, IL, USA). One-way analysis of variance (ANOVA) and Tukey’s comparison tests were employed to identify significant differences between the groups. Statistical significance was determined at a *p*-value of less than 0.05.

## 3. Results

### 3.1. Flexural Strength, Elastic Modulus, and Work-of-Fracture

**Figure 1:**
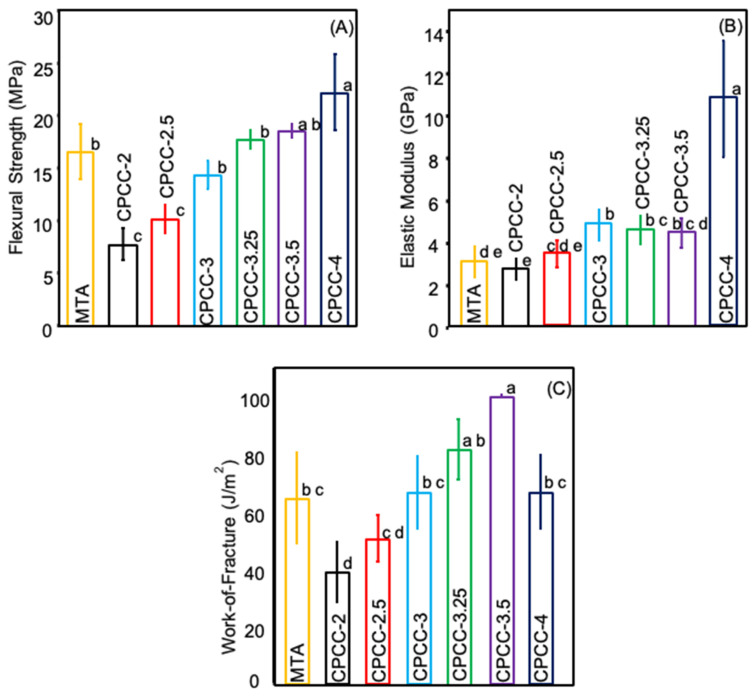
(**A**) Flexural strength, (**B**) elastic modulus, and (**C**) work-of-fracture of CPCC at various powder/liquid (P:L) formulations compared to MTA. An increased P:L ratio yields increased strength. Values with different letters indicate significant differences (*p* < 0.05).

Flexural strength, elastic modulus, and work-of-fracture results are shown in Figure 1 (mean ± SD; n = 6). CPCC-4 (22.2± 3.6) MPa demonstrated the highest flexural strength (*p* < 0.05). Meanwhile, CPCC-3 (14.3 ± 1.3) MPa, CPCC-3.25 (17.7 ± 0.9) MPa, and CPCC-3.5 (18.5 ± 0.6) MPa exhibited comparable strength to the MTA group (16.5 ± 2.6) MPa, (*p* > 0.05). The lowest values were observed with CPCC-2 (7.7 ± 1.5) MPa and CPCC-2.5 (10.1 ± 1.4) MPa (*p* < 0.05).

CPCC-4, CPCC-3.25, and CPCC-3 exhibited significantly higher elastic modulus values at (10.8 ± 2.76) GPa, (4.57 ± 0.68) GPa and (4.84 ± 0.72) GPa, respectively, compared to MTA (3.06 ± 0.74) GPa. In contrast, CPCC-2 (2.71 ± 0.48) GPa demonstrated a lower elastic modulus than MTA (3.06 ± 0.74) GPa (*p* < 0.05). However, no significant difference was observed between CPCC-2.5, CPCC-3.5, and MTA (*p* > 0.05).

No statistically significant differences were observed among CPCC-2.5, CPCC-3, CPCC-3.25, CPCC-4, and MTA regarding work-of-fracture values (*p* > 0.05). CPCC-2 displayed the lowest value, while CPCC-3 exhibited the highest value (*p* < 0.05).

### 3.2. Flowability

**Figure 2:**
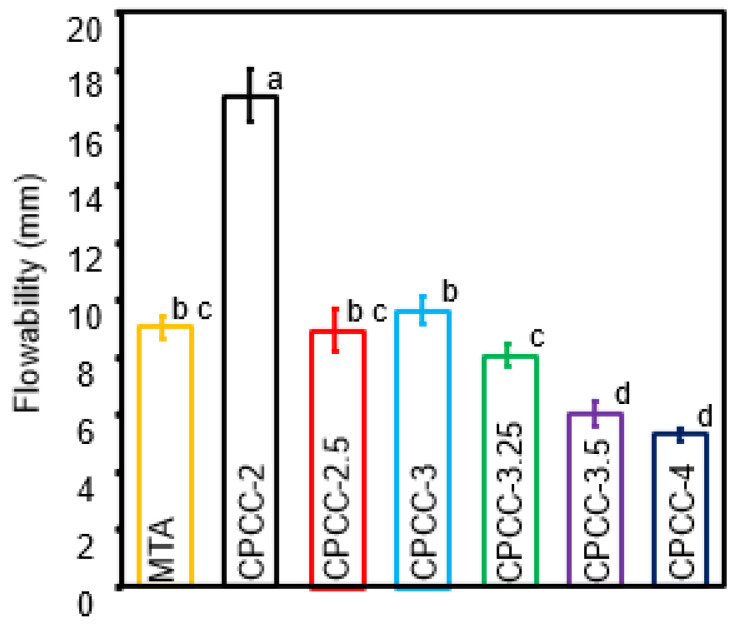
Flowability of CPCC at various P:L formulations compared to MTA. Flowability decreased as the P:L increased. Values with different letters indicate significant differences (*p* < 0.05).

Flowability results are depicted in Figure 2. The flowability of CPCC-2.5, CPCC-3, and CPCC-3.25 was similar to MTA at (8.93 ± 0.74) mm, (9.63 ± 0.48) mm, (8.05 ± 0.42) mm, and (9 ± 0.41) mm, respectively (*p* > 0.05). CPCC-2 exhibited the highest flowability at (17.05 ± 0.91) mm, whereas CPCC-3.5 and CPCC-4 were associated with the lowest flowability at (5.99 ± 0.45) mm and (5.31 ± 0.24) mm (*p* < 0.05).

### 3.3. Setting Time

**Figure 3:**
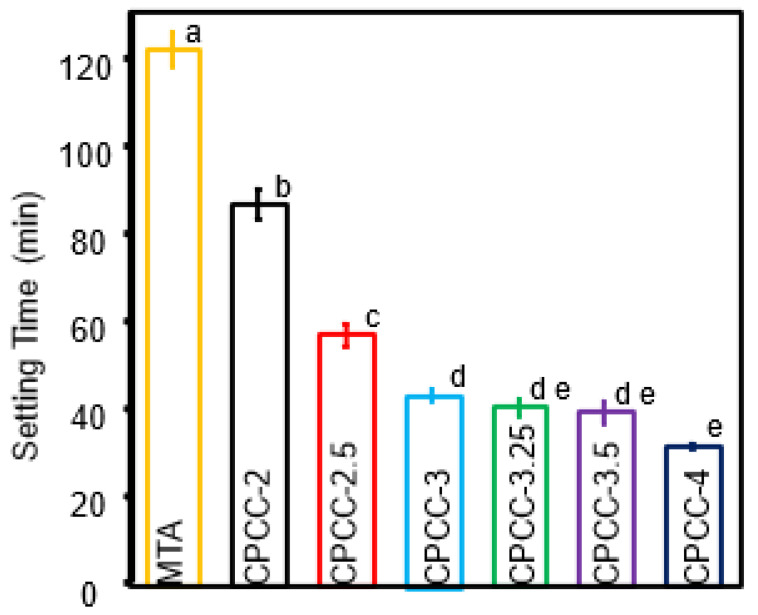
Setting time of CPCC at various P:L formulations compared to MTA. An increase in the P:L ratio led to a shorter setting time. Values with different letters indicate significant differences (*p* < 0.05).

Setting time data is shown in Figure 3. All CPCC formulations exhibited significantly shorter setting times compared to MTA. The longest setting time was observed with MTA (123 ± 4.2) min, while the shortest setting times were associated with CPCC-3.25 (41.5 ± 2.1) min, CPCC-3.5 (40.3 ± 2.5) min, and CPCC-4 (32.5 ± 0.7) min.

### 3.4. Metformin Release

**Figure 4:**
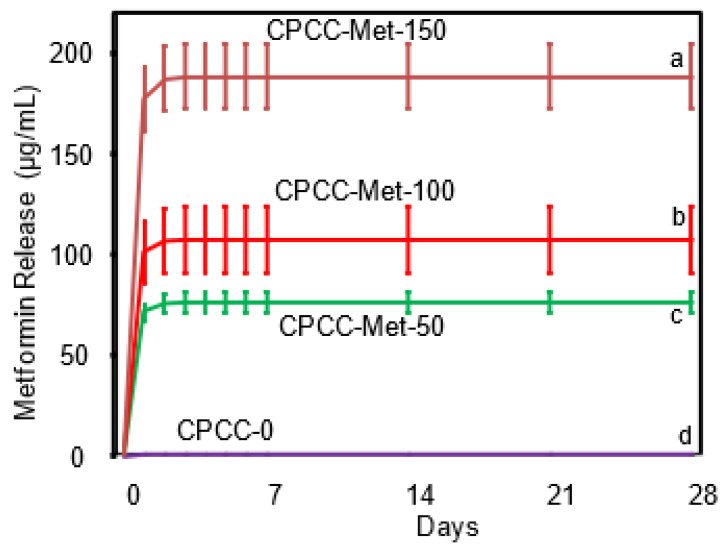
Measurement of released metformin from CPCC disks containing 0, 50, 100, and 150 µg metformin over 28 d. The amount of released metformin corresponds proportionally to its concentration. Values with different letters indicate significant differences (*p* < 0.05).

Metformin release data are illustrated in Figure 4. The release of metformin was directly proportional to the concentration of metformin loaded into the scaffolds. At 28 d, CPCC-Met-150 released (187.43 ± 15.99) µg/mL, CPC-Met-100 at (105.97 ± 16.44) µg/mL, CPCC-Met-50 at (74.91 ± 5.02) µg/mL, and CPCC-0 exhibiting no release (*p* < 0.05).

### 3.5. Cell Viability and Proliferation

**Figure 5:**
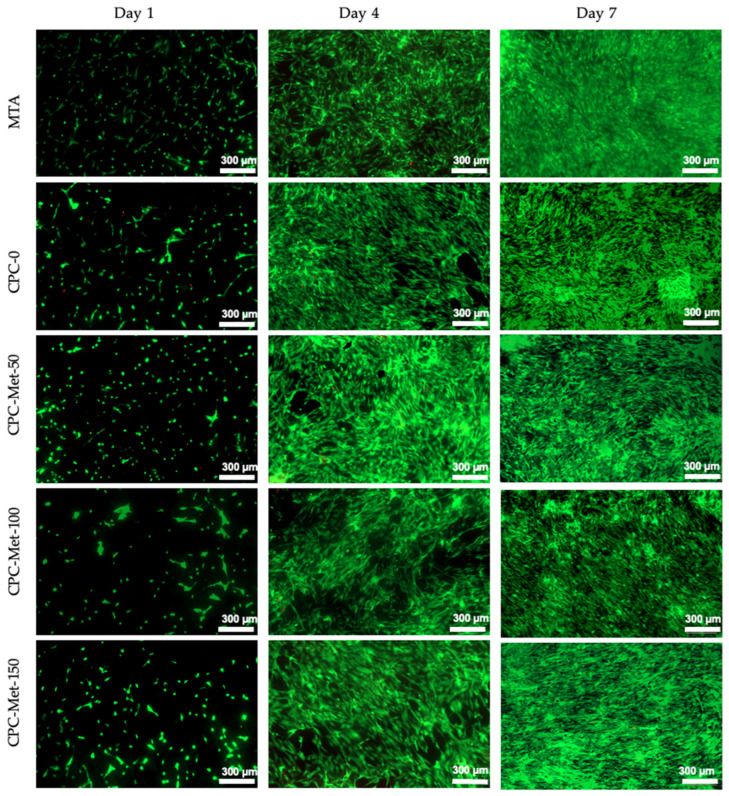
Representative fluorescence images of hDPSCs stained with live/dead stains on MTA and CPCC and CPCC-Met groups, with live cells stained green and dead cells shown in red. The hDPSCs were successfully attached to CPCC and CPCC-Met groups similar to MTA. Therefore, metformin can be delivered via CPCC pulp-capping material into the pulp to promote dentin repair without adversely affecting cell viability and attachment.

Figure 5 shows representative fluorescence images of hDPSCs stained with a live/dead assay on the MTA, CPCC-0, and CPCC-Met groups at 1 d, 4 d, and 7 d. The live hDPSCs, stained green, exhibited successful attachment with uniform coverage on both CPCC and CPCC-Met comparable to MTA. Conversely, the presence of dead hDPSCs, stained red, was minimal across all experimental groups, underscoring the excellent biocompatibility of the CPCC-based material.

**Figure 6:**
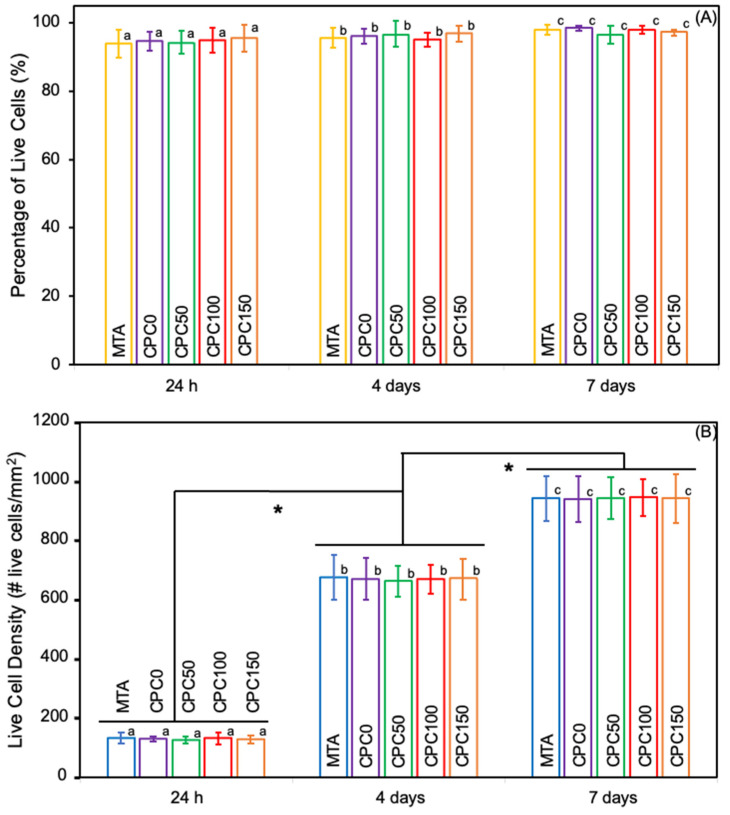
(**A**) Percentage of live cells and (**B**) live cell density per mm^2^ at 1 d, 4 d, and 7 d. The cell live/dead assay demonstrated that CPCC and CPCC-Met formulations had excellent cell viability (>93%) similar to MTA. Values with different letters indicate significant differences, and * denotes significant differences between time points in (**B**).

Figure 6A,B depicts the percentage of live cells and their density at 1 d, 4 d, and 7 d. Across all groups, the density of live cells exhibited a significant increase over time, indicating active cell proliferation (*p* < 0.05). There were no significant differences in either cell density or viability between the groups on any of these time points (*p* > 0.05). The percentage of live cells remained consistently high (>93% on day one, >95% on day four, and ≈98% on day seven), with no significant differences observed between all groups, highlighting excellent viability across all experimental conditions.

### 3.6. SEM

**Figure 7:**
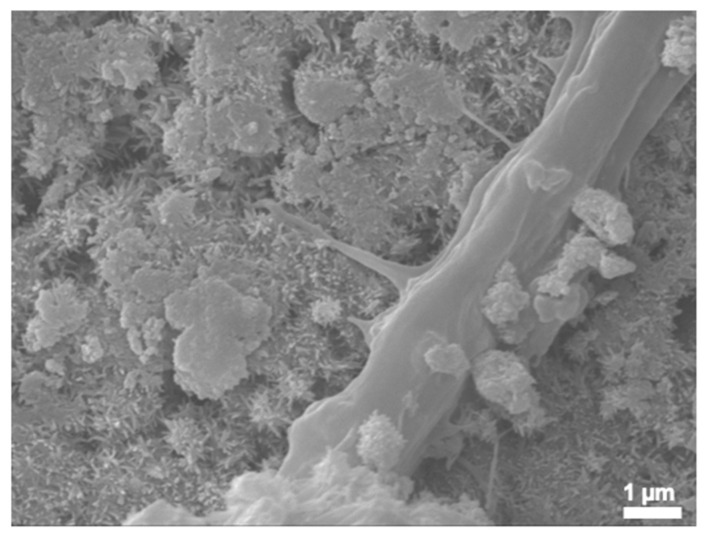
Nanostructured CaP showing hydroxyapatite in CPCC.

**Figure 8:**
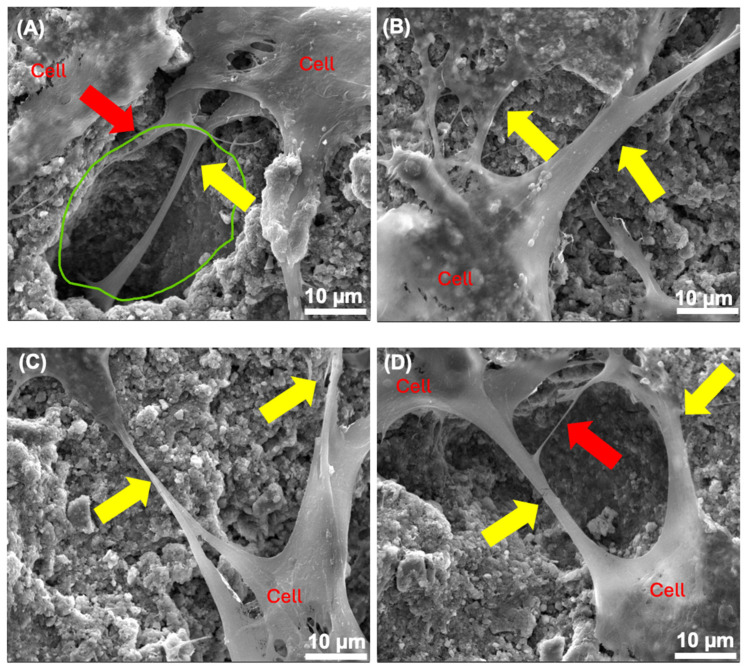
hDPSCs on CPCC-Met disks at different metformin concentrations. (**A**) 0 µg, (**B**) 50 µg, (**C**) 100 µg, (**D**) 150 µg. Yellow arrow: cell–cell junctions, red arrow: secondary extensions, and green circle: CPCC pore.

Figure 7 and Figure 8 show the SEM micrograph of CPCC and CPCC-Met with and without cells at 24 h. The CPCC disk surface had apparent pores and a well-formed hydroxyapatite crystal nanostructure (Figure 7 and Figure 8). The cell body had a diameter of approximately 20 µm and exhibited cytoplasmic extensions (pseudopodia) that extended up to 60 µm in length. Furthermore, cell–cell junctions (shown by yellow arrows in Figure 8A–D) were also established. Secondary extensions were observed around (A) and (D), as shown by the red arrows. They were firmly attached to the cement surfaces. Uniform cell spreading and attachment were observed on all specimens.

## 4. Discussion

The present study developed a novel self-setting CPCC-Met to be used as a direct pulp capping material for the first time. These formulations demonstrated enhanced physico-mechanical properties at a 3.25:1 ratio, with a threefold reduction in setting time compared to MTA. The metformin was successfully released from the cement matrix into the medium. The attachment and proliferation of hDPSCs on the disks were excellent in all experimental groups as observed in both live/dead staining and SEM images, indicating that the incorporation of metformin did not alter the material’s biocompatibility.

In recent years, modifications of MTA compositions have yielded to the development of newer materials such as Biodentine and NeoMTA as alternatives to ProRoot MTA [35]. These materials demonstrate improved handling, shorter setting times, and enhanced mechanical properties while maintaining high clinical success rates [36,37]. However, these materials remain expensive and their exothermic setting mechanism limits their potential as drug carriers [38]. Although our study focused on comparing CPCC with MTA, future work will include comparisons with modern bioceramic alternatives.

The mechanical properties of CPC depend mainly on its microstructure, which can be altered by different fabrication methods. These methods include variations in the molar ratio between TTCP and DCPA, different P:L ratio, and incorporating different additives. Mixing an equimolar ratio of TTCP and DCPA yielded the highest HA formation. Altering this ratio, however, can lead to the formation of other calcium phosphate phases, such as brushite [39]. During the setting phase of CPC, a network of elongated apatite crystals is formed, which is responsible for the material’s mechanical properties [17]. When the P:L ratio is increased, the formed apatite is closely packed and dense, thus improving the flexural strength and elastic modulus. Furthermore, the choice of liquid in the mixture plays a critical role. The use of chitosan, for instance, promotes a more cohesive and dense matrix by fusing HA particles together, thereby enhancing the material’s ability to withstand mechanical stress [40]. However, the exact correlation with work-of-fracture is complex, as this property is not solely dependent on material density; factors such as the ability to hinder crack propagation or a potential shift in material behavior towards brittleness may also play a role [17].

The flowability of CPCC is closely related to the P:L ratio; as the liquid content increases, the flowability increases, and vice versa. In this study, the P:L ratio ranged from 2:1 to 4:1 because ratios exceeding 4:1 resulted in an excessively dry paste for mixing and molding, while ratios below 2:1 yielded specimens with diminished strength. By adjusting the P:L ratio within this range, the flowability can be tailored to meet the specific requirements of the desired clinical application. For instance, the material must exhibit sufficient flowability to ensure optimal coverage and conform to the irregularities when used as a direct pulp capping.

MTA’s main drawbacks are its long setting time and high cost [7,41]. Its setting reaction relies primarily on hydration processes [41]. When mixed with water, MTA powder forms calcium hydroxide (CH) and calcium silicate hydrate, which eventually become a poorly crystallized, porous gel. The calcium silicate content decreases due to the formation of a calcium precipitate. This precipitate produces CH, leading to MTA’s high alkalinity [10]. Multiple manufacturers claimed that the MTA setting time could be significantly reduced. However, those claims are misleading as they do not consider the hydration rate, which is detrimental to the MTA setting [41]. The hydration rate of MTA refers to the speed at which the material reacts with water to form its set structure, including the formation of calcium silicate hydrate and CH [10]. This results in a loose, sandy mixture with no thickness that is difficult to handle clinically. Therefore, a short setting time is crucial when designing a material to replace MTA. The setting time could be shortened by increasing particle wettability via reducing powder particle size or implementing a higher P:L ratio [42]. CPC setting time decreases significantly at body temperature, enhancing the material’s practicality for clinical applications [43]. The type of liquid and its pH also affect the setting time. CPC’s setting time can be reduced when mixed with chitosan rather than water. When CPC reacts with an acidic chitosan solution, such as chitosan-malate solution, the initial low pH enhances the dissolution of the calcium phosphate components, accelerating the setting time. As the setting reaction progresses, the formation of CH raises the pH. This increase in pH, resulting from the setting reaction, causes the CPC paste to solidify and form a hard bulk faster. Thus, the initial acidity of the chitosan solution accelerates the initial solidification of the CPC, followed by further chemical interactions involving TTCP and DCPA to create HA [40].

One critical property of pulp-capping agents is their ability to set in the presence of moisture, given the highly hydrated nature of exposed pulp tissues and the potential for minor persistent bleeding during treatment [44]. While CPCC has demonstrated promising physico-mechanical properties, further studies are required to evaluate its sealing ability and performance under clinically relevant moist conditions, which are vital for long-term success.

Unlike MTA, the CPC setting reaction is non-exothermic. This thermal stability allows CPC to serve as an excellent vehicle for local drug delivery, since there is minimal risk of thermal degradation of the therapeutic agent. CPC has been utilized as a carrier for antibiotics, non-steroidal anti-inflammatories, anti-osteoporotic agents, and various other drugs [13,45].The drug can be added to the liquid, powder, or pre-set CPC via immersion or dropwise addition. Incorporating the drug into CPC liquid enables a more homogeneous distribution throughout the cement matrix. However, some drugs may degrade or lose efficacy when mixed directly with liquid. In the case of metformin, the slight acidity of chitosan has been shown to help stabilize it without losing its therapeutic effect upon release [27,46,47].

Metformin is commonly used as an oral antidiabetic to treat type 2 diabetes mellitus. It has a long history of excellent safety and tolerability and is more stable when mixed in an acidic medium [22]. In our prior study, CPC incorporating up to 50 μg of metformin enhanced the proliferation of hDPSCs [48,49]. Additionally, the efficacy of metformin is primarily determined by its dosage, rather than the frequency of administration [49]. In this study, the data suggest incorporating up to 150 µg of metformin did not affect the cell attachment or viability. Hence, metformin can be delivered via CPCC pulp-capping material into the pulp without compromising cell viability. Metformin was mixed with the liquid to enhance the homogeneity and distribution of the drug. It exhibited a significant initial burst release corresponding to the concentration initially loaded into the CPCC, likely attributable to the material’s porosity. However, the material porosity may change clinically over time due to the formation of an apatite layer on the CPC surface that slows the release and extends the release period, which aids in a longer sustained release—a phenomenon termed the membrane effect [13].

SEM showed the formation of the CPC end product HA, which is the building block for mineralized tissue. Mixing an equimolar ratio of TTCP and DCPA, as well as setting in a pH greater than 4.2—which is typically the normal body pH—results in the end product of CPC being HA [50]. HA is the main component of the inorganic part of teeth and bone and constitutes at least 70% of dentin [51]. It is widely accepted that a pore size of at least 50–100 μm is necessary for the ingrowth of mineralized tissue [52]. Being intrinsically microporous, CPC allows the ingrowth of mineralized tissue. The pores provide space for odontoblasts to adhere, proliferate, and mature, which is essential for regenerative tissue formation [52]. This is evident from the observation of pseudopodia extensions formation on the CPC. Pseudopodia are dynamic, protruding extensions of the plasma membrane that facilitate cell movement and interaction with the substratum.

While cell adhesion and proliferation observed in CPCC-met disks is encouraging, it does not directly confirm the induction of odontoblast-like cells or the formation of a dentinal bridge. This is a limitation, as successful pulp capping relies on the formation of a complete dentinal bridge. Future studies should assess odontoblast differentiation markers, such as DSPP expression, and histological evaluation of dentinal bridge formation in vivo to validate the clinical potential of CPCC-Met.

The novel CPCC-Met in this study can be tailored to meet the desired clinical application by adjusting the P:L ratios. It demonstrated improved properties, including releasing metformin and forming HA. Metformin did not alter cell adhesion and proliferation on the disk surfaces, as confirmed by live/dead assay and SEM. Therefore, the CPCC-Met formulations described here can substantially reduce clinical time and cost when used for direct pulp capping. This preliminary study concentrates on in vitro assessments to determine the fundamental features and biological responses of the novel CPCC-Met, while subsequent research will progress to animal models to evaluate the system’s efficacy in a more complex biological context.

## 5. Conclusions

The novel CPCC-Met demonstrated excellent physical and mechanical properties matching those of MTA, while cutting the cement setting time by over an hour. All groups had excellent hDPSC adherence and viability. With much shorter setting time and lower cost, the novel CPCC-Met holds promise as an effective pulp capping material and carrier for local release of metformin to heal the pulp.

## Data Availability

The data presented in this study are available upon request from the corresponding authors.
